# Mood Shapes Reliance on Syntactic and Semantic Cues in Sentence Comprehension

**DOI:** 10.3390/bs15111591

**Published:** 2025-11-20

**Authors:** Xinmiao Liu, Shengqi Wu, Xiaoli Wang

**Affiliations:** 1 National Research Centre for Foreign Language Education, Beijing Foreign Studies University, Beijing 100089, China; liuxinmiao@bfsu.edu.cn; 2 School of English for Specific Purposes, Beijing Foreign Studies University, Beijing 100089, China; 3 School of Foreign Languages, Renmin University of China, Beijing 100872, China

**Keywords:** mood, sentence comprehension, syntax, semantics

## Abstract

The linguistic system relies on both syntactic and semantic cues to derive the meaning of sentences. Although this process is shaped by cognitive factors, little is known about how mood influences reliance on these cues and whether such effects are moderated by cognitive control. This study examined how positive and negative mood influence the use of syntactic and semantic information and whether inhibitory control and working memory moderate these effects. A sentence judgement task was administered among participants with high and low valence. The semantic plausibility and syntactic consistency of the experimental stimuli were manipulated. The results revealed a significant interaction between syntactic consistency and semantic plausibility in negative moods. In positive moods, cue use was more independent. Inhibitory control was found to influence the interaction between syntactic consistency and semantic plausibility in negative moods but not in positive moods. Working memory had no significant influence on syntactic consistency or plausibility in either mood condition. These findings provide valuable insights into the interplay between affect and cognition in shaping sentence comprehension.

## 1. Introduction

Both syntactic and semantic cues provide essential information with which to construct meaning in sentence comprehension. Syntactic cues such as word order or case-marking help to identify grammatical relations between words. Semantic cues, on the other hand, constitute meaning-bearing information that facilitates sentence comprehension. These cues involve linguistic knowledge, visual symbols, and the comprehenders’ life experiences. For instance, in comprehending the sentence “*The boy that bit the dog ran away*”, we can learn from syntactic rules that the noun *dog* is the patient of *bit*, and *boy* is the agent of *bit*. However, according to our world knowledge, *dog* is a more plausible agent of *bit*, and *boy* is the more plausible patient. Syntactic analysis and semantic heuristics provide two distinct routes to understanding sentences (Forster & Olbrei, 1973). Comprehenders may rely on the two routes to different extents. For example, in noisy surroundings where auditory input is compromised, we tend to rely more on semantic cues to understand speech. According to the good-enough processing theory (Ferreira et al., 2002; Ferreira, 2003; Ferreira & Patson, 2007), sentence processing isn’t always thorough and complete. Instead of fully working out all grammatical details, we sometimes rely on semantic shortcuts to understand sentences quickly. Semantic heuristics is an economical processing strategy that enables us to efficiently extract meaning from linguistic inputs without overtaxing our cognitive resources.

Previous studies have found that there might be individual differences in the reliance on semantic or syntactic cues. For example, comprehenders with low working memory (WM) capacity tend to rely more on semantic cues in sentence comprehension (Christianson et al., 2006; Stella & Engelhardt, 2022; Liu, 2024). Christianson et al. (2006) examined the processing of garden-path sentences in younger and older adults. They found that older adults were more likely to accept transitive interpretations for optionally transitive verbs compared with younger adults due to reliance on semantic inferences and reduced working memory. Older adults performed only at chance levels in interpreting reflexive absolute transitive verbs in garden-path sentences, with accuracy linked to WM capacity. The findings suggest incomplete syntactic reanalysis is more common in older adults due to their memory limitations. In a self-paced reading experiment, Liu (2024) found that older adults shifted their processing strategy toward semantics rather than syntax under high working memory burden, unlike their younger counterparts. These studies suggest that ageing and the accompanied working memory decline drive comprehenders to resort to semantic heuristics during sentence comprehension. However, most of these studies have examined merely the effect of WM on the use of syntactic or semantic cues. Little attention has been paid to the influence of other factors such as comprehenders’ mood states.

Individuals’ affective states affect various cognitive functions like reasoning, memorization, and inference. Sentence processing is a process of constructing the meaning of a sentence from visual or auditory inputs. To arrive at the correct understanding of a sentence, the human processor needs to temporally store certain words for later integration, make inferences about upcoming words, or suppress irrelevant information. As this process involves memorization, inference, and reasoning, sentence comprehension may also be influenced by mood conditions. Several studies have explored how mood affects semantic processing (Federmeier et al., 2001; Chwilla et al., 2011; Vissers et al., 2013) or syntactic processing (Chwilla, 2022; Verhees et al., 2015). Federmeier et al. (2001) investigated how transient mood affects semantic memory using event-related potentials. Participants in positive or neutral mood conditions were instructed to read sentences ending with expected, within-category unexpected, or between-category unexpected words. The results revealed semantic category effects in a neutral mood, with the smallest N400 amplitude observed for expected words and smaller amplitudes observed for within-category unexpected words, but positive mood eliminated differences between the two unexpected word types. These findings suggest that positive mood facilitates the processing of distantly related words. Chwilla et al. (2011) tested how mood influences semantic processing using sentences that were either highly plausible (e.g., *The pillows are stuffed with feathers*) or implausible (e.g., *The pillows are stuffed with books*). They found that the N400 effect was reduced in negative moods. Studies about the effect of mood on syntactic process are relatively scare (Chwilla, 2022; Verhees et al., 2015). For instance, Vissers et al. (2010), using ERP methods, observed that P600 was more widely distributed when participants were in a positive mood, but its distribution reduced under negative mood conditions. Verhees et al. (2015) found that when attention was devoted to syntactic features, a sad mood was associated with a reduced P600. The findings suggest that mood and attentional focus interact to influence syntactic parsing. However, no significant effect of positive or negative mood on syntactic parsing was detected in Van Berkum et al. (2013)’s study.

Previous research has examined how mood may influence syntactic or semantic processing. The findings are mixed, and these processes have mostly been investigated in isolation. It is still not clear whether individuals in positive and negative affective states differentially use syntactic and semantic cues to understand sentences. Since mood influences cognitive processing styles (Schwarz et al., 1991; Schwarz, 2002), it may shape the extent to which different types of linguistic information are utilized. Bless et al. (1992) reported that participants experiencing positive mood were less detail-oriented than those in a negative mood in processing persuasive communication, a finding that highlights the role of mood in language communication. However, the specific ways in which mood might affect the use of syntactic and semantic cues remain unclear. Many studies suggest that mood’s effect on language processing is modulated by cognitive factors such as attention (Verhees et al., 2015). Given the intertwined nature of emotion and cognition, individuals’ general cognitive abilities such as inhibitory control may also modulate the influence of mood. Inhibitory control has been defined as the capacity to regulate one’s attention, actions, and emotions so as to resist impulses or distracting external stimuli (Diamond, 2013). In language comprehension, inhibitory control can suppress contextually inappropriate information by keeping it away from working memory or deactivating it if it is in the memory (Hasher & Zacks, 1988). Earlier research has shown that positive affect is linked to reduced inhibition and broader processing styles, whereas negative affect can increase inhibition and analytic processing (Fredrickson, 2001; Gasper & Clore, 2002; Isen, 2000; Katzir et al., 2010). These differences may influence how comprehenders prioritize syntactic or semantic cues. In addition, since WM has a significant impact on sentence processing (Christianson et al., 2006), it may also be a crucial modulating factor of the effect of mood. So far, no empirical studies have explored the modulating role of inhibitory control and WM in mood’s effect on the use of semantic and syntactic cues in sentence comprehension.

This study aims to investigate how comprehenders in different mood conditions use syntactic and semantic cues differently in sentence processing and how such mood effects are influenced by inhibitory control and working memory. This investigation can help us understand the adaptive nature of sentence processing under different affective and cognitive conditions. Syntactic processing in sentence comprehension aligns with an analytic processing mode in that both emphasize structural relations. Semantic processing resembles heuristic reasoning as both rely on prior knowledge (Andrews et al., 2017). Therefore, this study draws on reasoning research to explore how positive versus negative mood shapes cue use in sentence processing. According to the feelings-as-information theory, pleasant affect promotes a heuristic style of thinking, whereas unpleasant affect often leads to analytical thinking (Beukeboom & Semin, 2002; Schwarz, 2001; Storbeck & Clore, 2008). Therefore, comprehenders in a positive affective state may rely more on semantic cues in sentence processing, whereas those in a negative mood might prefer syntactic cues. Such mood effects may be modulated by inhibitory control and working memory due to the interaction between cognitive and affective factors. Alternatively, the broaden-and-build theory suggests that positive affect fosters cognitive flexibility and broadens cognitive scope (Fredrickson, 1998, 2001). Thus, comprehenders in a positive mood might shift more flexibly between the use of semantic and syntactic cues, possibly resulting in separate main effects of each cue type. As negative affect narrows cognitive scope (Fredrickson & Branigan, 2005), comprehenders may rely on more rigid processing, prioritizing either semantic or syntactic cues depending on contextual demands. This pattern may manifest as an interaction between semantic and syntactic cues. To test these hypotheses, we implemented a sentence judgment task in which participants evaluated sentences varying in semantic plausibility and syntactic consistency under high- and low-valence conditions. This study can further our understanding of how affective states interact with cognitive processes to shape language comprehension and thus have important implications for models of sentence processing and inform strategies for effective communication in emotionally charged contexts.

## 2. Materials and Methods

### 2.1. Participants

A total of 75 undergraduate students from Chinese universities took part in the study. They were assigned randomly to two groups (positive versus negative), with 37 in the positive-valence group and 38 participants in the negative-valence group. All participants spoke Chinese as their native language. Data from one individual in the positive-valence group were omitted as they were incomplete. No statistically significant group difference was found in gender ratio, *χ*^2^ = 0.002, *df* = 1, *p* = 0.964, age, *t*(72) = 0.01, *p* = 0.993, and year of education, *t*(72) = 0.74, *p* = 0.461. Demographic details are summarized in Table 1.

### 2.2. Materials

Participants completed a mood induction task, a sentence comprehension task, a Stroop task and a backward digit span test. Film clips were used to induce positive and negative affective states. The materials were selected from the standardized database of Chinese movie clips (Ge et al., 2019) according to their normative ratings. Positive and negative movie clips were used in our experiment with high/low valence ratings and matched arousal scores. For the positive movie clips, we used one clip from Love on Delivery (mean valence = 7.71, SD = 1.18) and one from Singing When We Are Young (mean valence = 5.91, SD = 2.16). For negative clips, we used a clip from City of Life and Death (mean valence = 1.26, SD = 0.67) (Ge et al., 2019). Participants’ affective states were measured with the Self-Assessment Manikin (SAM; Bradley & Lang, 1994), a 9-point pictorial scale widely used in psychology research. It consisted of three items measuring valence, arousal and dominance. The scale is a set of manikin figures that depict graded levels of each dimension, allowing participants to indicate their psychological state without words. Participants were asked to choose the manikin that can best reflect their feelings.

This study adopted a sentence judgment task using the conclusion evaluation technique. This technique has been used in prior research to explore semantic and syntactic processing (Andrews et al., 2017; Liu, 2024). We use endorsement in premise-conclusion judgments as a post-interpretive measure of sentence comprehension, following established psycholinguistic practice (e.g., Andrews et al., 2017; Bornkessel et al., 2005; Ye & Zhou, 2009). Previous research has demonstrated consistent alignment between online processing measures, such as reading times, and offline endorsement patterns. For example, garden path sentences, which initially lead to parsing errors, produce online processing difficulties that correspond with offline misinterpretations (e.g., Slattery et al., 2013). Studies integrating online and offline methods further indicate that *good-enough* comprehension outcomes are evident in both real-time processing and subsequent judgments (e.g., Fujita & Cunnings, 2020; Qian et al., 2018). Event-related potential (ERP) studies have demonstrated that semantic attraction in sentences involving syntax-semantics conflicts elicits patterns of N400 and P600 components, which reflect parallel competition between a heuristic route driven by semantic cues and an analytic route guided by syntactic rules (e.g., Kim & Osterhout, 2005; Kim & Sikos, 2011; for reviews, see Leckey & Federmeier, 2020). Crucially, the direction of these online neural signals tends to align with participants’ subsequent endorsement decisions in offline comprehension judgments (Kos et al., 2010). Thus, while our endorsement measure is post-interpretive, it reflects mechanisms consistent with those observed in online sentence processing. The conclusion evaluation task typically involves *premise* and *conclusion*, and participants are instructed to judge whether the conclusion follows logically from the premise. The task in the present study included 56 sets of sentences. Each set consisted of a sentence (*premise*) and a statement (*conclusion*). The sentences were Mandarin relative clauses. Relative clauses were chosen as experimental stimuli because this structure allows for the manipulation of both syntactic and semantic cues while controlling for lexical confounds. We created a statement for each sentence. The manipulation involves the relationship between the sentence and the statement. The statement either matches or violates the correct syntactic interpretation of the sentence. Syntactic consistency indicated whether the statement preserved the predicate-argument mapping licensed by the correct parse of the sentence. Conclusions were considered as consistent when they matched this mapping and inconsistent when they did not. Half of the conclusions were syntactically consistent, and the remainder were not. Semantic plausibility referred to whether, given the entities and relations introduced by the premise, the statement described a likely event in world knowledge (plausible vs. implausible). Fifty percent of the statements were plausible, and the rest were not. This resulted in four experimental conditions including syntactically consistent and semantically plausible (CP), syntactically inconsistent and semantically implausible (IncImp), consistent but implausible (CImp), and plausible but inconsistent (IncP). Sample stimuli are shown in Table 2. Each condition includes 14 sets of sentences. 56 fillers with diverse sentence structures were also included.

A norming study was administered before the main experiment to check the validity of plausibility manipulation. A group of 20 subjects were invited to rate the semantic plausibility of the sentences and statements. They did not participate in the main experiment. Sentence plausibility was rated using a five-point Likert scale ranging from 1 (very implausible) to 5 (very plausible). The results revealed that the ratings for plausible items are significantly higher than that for implausible items (*p* < 0.001). No significant differences were found between CP (M = 4.26, SD = 1.07) and IncP (M = 4.20, SD = 1.14), *t* = 1.18, *p* = 0.234. Similarly, no significant differences were found between CImp (M = 2.05, SD = 1.22) and IncImp (M = 2.10, SD = 1.28), *t* = −0.98, *p* = 0.328. These results indicate that the plausibility manipulation was not influenced by syntactic structure. Thus, any differential performance across these conditions in the main task can be attributed to differences in semantic plausibility rather than syntactic confounds. This supports the validity of the plausibility manipulation in the present study.

### 2.3. Procedures

The experiment was designed and implemented with the Gorilla Experiment Builder (Anwyl-Irvine et al., 2020), a web-based platform for running behavioral experiments. Participants completed tasks online using their personal computers. First, they were randomly assigned to a positive or negative valence condition. They were instructed to watch the selected film clips on computer screens and immerse themselves in the scenes. To promote engagement, they were asked to respond to a brief question about each clip. Their affective states were evaluated using the Self-Assessment Manikin scale (SAM; Bradley & Lang, 1994) before induction, immediately afterwards, and again upon completion of the experiment.

Following the mood induction, participants performed the sentence judgement task. Sentences were presented at the computer screen using a noncumulative self-paced reading paradigm. Participants pressed the spacebar to advance through the premise sentence word by word. With each word appearing at the screen, the previous word was masked from view. Upon reading the sentence, a statement showed up on the screen as a whole. Participants read the statement and judged whether it followed logically from the previous sentence. On the response screen they saw two options: “Yes” at the bottom-left and “No” at the bottom-right. They responded by pressing the left arrow key (←) for “Yes” response or the right arrow key (→) for “No”. They completed two practice trials to familiarize with the experimental procedures.

After the sentence judgement task, the Stroop color word task was administered to measure inhibitory control. This test has been widely used to measure inhibitory control and cognitive interference (Stroop, 1935). The task required participants to identify the color of words and ignore their meaning. Four colors were used: red, yellow, blue and green. Participants responded by pressing one of the four keys: ‘O’ for blue, ‘W’ for yellow, ‘Q’ for red, and ‘P’ for green. The task was divided into two phases: a practice session and the main experiment. During the practice, participants read instructions, and completed 15 trials. Feedbacks were provided after each trial. The main task includes 50 trials with no feedbacks. The task included congruent trials, where the color of words matched word meaning and incongruent trials, where word colors did not match word meaning. Participants were asked to give rapid responses. Response times and accuracy were recorded. Stroop effect is defined as the differences in RTs between incongruent stimuli and congruent stimuli for correctly responded items. Larger Stroop scores indicate greater interference and weaker inhibitory control, and lower Stroop scores reflect better ability to suppress irrelevant information. The Stroop scores are centralized and standardized before analysis.

Finally, the backward digit span task was administered to assess working memory capacity. In each trial, a sequence of digits was displayed one by one, and participants were required to retain them in memory. Once the sequence ended, they typed the numbers in reverse order. The test contained 12 trials. The trials began with three digits and increased gradually to eight digits. Participants were asked to respond as fast as they could. Their performance on the task was scored based on the total number of correct answers. The entire experiment lasted for about 40 min.

### 2.4. Data Analysis

Statistical analyses were conducted in R (Version 4.4.2; R Core Team, 2021). To examine the effects of mood states, syntactic consistency, semantic plausibility and inhibitory control, we employed a combination of statistical models tailored to the data structure. Binary endorsement responses were modeled with logistic mixed-effects models (lme4, Version 1.1-35.5; Bates et al., 2015). For reaction times (RTs) and response type relationships (syntax-based and semantics-based), we used generalized additive mixed models (GAMM) implemented via the mgcv package (Wood, 2025), allowing for non-linear smooth terms to capture temporal dynamics. Continuous variables including RTs and Stroop scores were centered and standardized before analysis. Post-hoc comparisons were performed to explore significant interaction using the emmeans package (Lenth, 2025). P-values for logistic mixed-effects models were calculated with the lmerTest package (Kuznetsova et al., 2017), and GAMM p-values were based on chi-square tests of smooth terms. All models included random intercepts for subjects and items.

## 3. Results

### 3.1. Descriptive Statistics

To evaluate task performance and data quality, we report descriptive statistics for accuracy rates and reaction times across groups and conditions. We excluded the data from the participants who had mean comprehension accuracy below 75% or completed less than 90% of trials. This resulted in the removal of one participant from the positive-valence group due to incomplete responses, leaving 36 participants in the positive-valence group and 38 in the negative-valence group. No participants were excluded for low accuracy. Mean accuracy and reaction times across group and conditions were summarzied in Table 3.

### 3.2. Valence Rating Analysis

**Figure 1:**
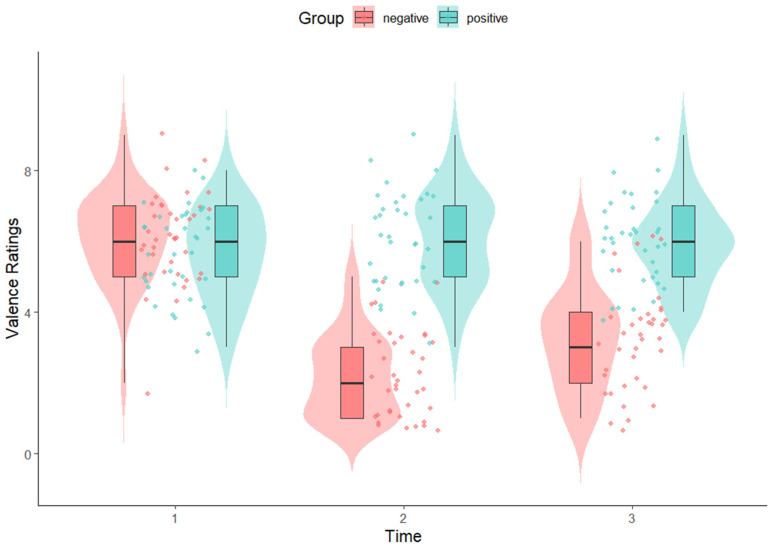
Mean valence ratings by group and time.

Participants’ affective states were assessed at three points: prior to the induction (Time 1), immediately after the induction (Time 2), and at the end of the experiment (Time 3). Mean valence ratings by the two groups were shown in Figure 1. To check whether the induction was valid, we fitted a mixed-effects model with group (negative vs. positive), time (1, 2, 3), and their interaction as fixed effects. At Time 1, no significant group difference in valence ratings was observed (*β* = −0.41, *SE* = 0.30, *t* = −1.36, *p* = 0.174). A signficant group difference was found in changes of valence ratings from Time 1 to 2, (*β* = 4.28, *SE* = 0.38, *t* = 11.05, *p* < 0.001), and from Time 1 to 3, (*β* = 3.09, *SE* = 0.38, *t* = 7.97, *p* < 0.001). In the negative group, valence ratings significantly declined from Time 1 to 2 (*p* < 0.001), then increased from Time 2 to 3 (*p* < 0.01). The ratings in the positive group did not differ between Time 1 and 2 (*p* = 0.465). Between-group comparisons revealed no significant difference in valence ratings at Time 1 (*p* = 0.178), but significant differences at Time 2 and 3 (*p*s < 0.001). This shows that the two groups did not differ in affective states before induction, but the positive group reported significantly higher valence than the negative group after induction and during the task. After induction, the mean ratings for the positive-valence group were significantly above the neutral threshold of five (*p* < 0.01), and those for the negative-valence group were significantly below it (*p* < 0.01). It should be pointed out that the positive-valence group did not show a significant change in ratings from baseline, likely due to already high baseline valence levels. This indicates that their positive affective state was likely pre-existing rather than induced by the movie clips. Therefore, the positive mood induction in this study was not effective. However, the mean ratings for the positive-valence group were significant above the neutral threshold of five (*p* < 0.01) and higher than those for the negative group, which enables us to make valid comparisons between the two groups based on the observed differences in valence.

### 3.3. Endorsement Analysis

We investigated the effects of plausibility (plausible vs. implausible) and consistency (consistent vs. inconsistent) on endorsement data across the two groups. Endorsement refers to “yes” responses to comprehension questions. It reflects post-interpretive judgement indexing the outcome of comprehension. In the conclusion evaluation paradigm, endorsement pattern provides interpretable indices of cue reliance. Previous studies have shown that the use of endorsement as the dependent variable can effectively isolate reliance on syntax vs. semantics in this paradigm (Andrews et al., 2017; Liu, 2024). We analyzed endorsement data rather than accuracy as our main interest was whether participants endorsed a syntactic or semantic interpretation of the sentences.

#### 3.3.1. Mood-Related Differences in the Effects of Consistency and Plausibility

A logistic mixed-effects model was fitted with group, syntactic consistency, semantic plausibility and all interactions as fixed factors. Results revealed a significant effect of plausibility (*β* = 1.70, *SE* = 0.35, *z* = 4.83, *p* < 0.001) and consistency (*β* = −5.30, *SE* = 0.25, *z* = −20.68, *p* < 0.001), indicating that participants endorsed plausible sentences more than implausible sentences, and syntactically consistent sentences more than inconsistent ones. Group differences were not statistically significant (*β* = 0.44, *SE* = 0.23, *z* = 1.92, *p* = 0.055), and the interaction with syntactic consistency was also nonsignificant (*β* = −0.42, *SE* = 0.33, *z* = −1.28, *p* = 0.199). The interaction between group and plausibility was significant (*β* = −1.04, *SE* = 0.43, *z* = −2.37, *p* < 0.05). Consistency and plausibility interacted significantly (*β* = −2.16, *SE* = 0.42, *z* = −5.09, *p* < 0.01), indicating that the effect of plausibility varied between consistency conditions.

**Figure 2:**
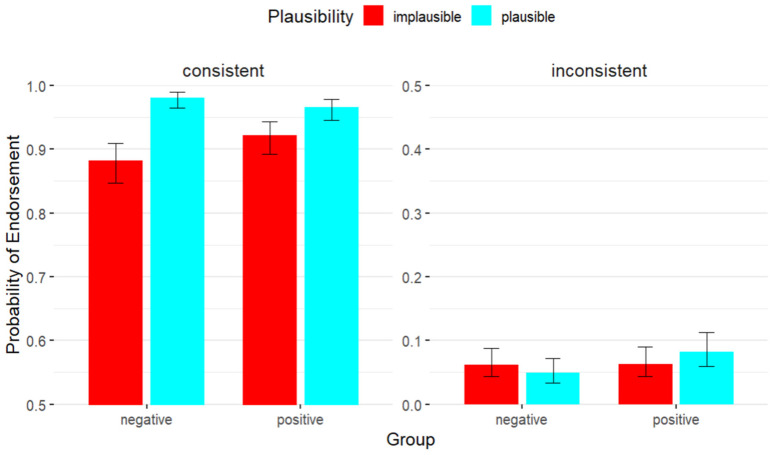
Probability of endorsement as a function of group, plausibility and consistency.

More importantly, a significant interaction was observed among group, plausibility and consistency (*β* = 1.55, *SE* = 0.56, *z* = 2.77, *p* < 0.01). Further analysis demonstrated that the relationship between consistency and plausibility was statistically significant in negative affective state (*p* < 0.001), but not positive affective state (*p* = 0.105). For syntactically consistent items, the difference in the plausibility effect between the negative and positive valence was significant (*p* < 0.05), with the plausibility effect stronger in participants with negative valence. For inconsistent sentences, the difference in the plausibility effect between groups was not significant (*p* = 0.239). As shown in Figure 2, the variation of plausibility effect across the two consistency conditions was greater in the negative-valence group.

To test whether the endorsement pattern reflected potential re-reading of experimental stimuli, we included reading times for premises and the conclusion statements as covariates in the mixed-effects model above. Reading times were log-transformed and z-scored within participant. A likelihood-ratio test showed that adding these two reading time covariates did not improve model fit (*χ*^2^ = 0.008, ΔAIC = 4.0, *p* = 0.996). The fixed effects for group, plausibility, consistency, and their interactions were unchanged in direction and significance. These results indicate that reading-time variance for premises and statements does not account for the endorsement patterns reported in the main analysis.

#### 3.3.2. The Influence of Inhibitory Control and Working Memory

We further explored the role of general cognitive factors including inhibitory control (IC) and working memory (WM) in the mood-by-language interactions. The IC data outside the range of ±2.5 standard deviations from the means were considered outliners and excluded from analysis. 2.63% of the data were excluded in the negative valence condition and 2.78% in the positive valence condition. The data were then standardized to facilitate interpretation. Logistic mixed-effects modeling was employed to assess the influence of group, semantic plausibility, syntactic consistency, IC, and their interactions on responses. The main effect of IC was not significant (*β* = 0.25, *SE* = 0.16, *z* = 1.53, *p* = 0.125). However, IC interacted significantly with semantic plausibility (*β* = −0.85, *SE* = 0.40, *z* = −2.12, *p* < 0.05) and syntactic consistency (*β* = −0.71, *SE* = 0.24, *z* = −2.89, *p* < 0.05), indicating that IC influenced the use of syntactic and semantic cues. The interaction between IC, group, and semantic plausibility did not reach significance (*β* = 0.81, *SE* = 0.51, *z* = 1.58, *p* = 0.114), whereas a significant three-way interaction emerged between IC, group, and syntactic consistency (*β* = 0.88, *SE* = 0.37, *z* = 2.38, *p* < 0.05). This suggests a group-specific influence of IC on syntactic process. The interaction between semantic plausibility, syntactic consistency, and IC was significant (*β* = 1.44, *SE* = 0.49, *z* = 2.92, *p* < 0.05). The four-way interaction involving IC, group, semantic plausibility, and syntactic consistency was also significant (*β* = −1.57, *SE* = 0.65, *z* = −2.41, *p* < 0.05), highlighting complex interdependencies among these variables. Given the complexity of the four-way interaction, separate analyses were carried out for each group to explore the different processing mechanisms.

In the positive valence condition, the main effect of plausibility (*β* = 0.97, *SE* = 0.30, *z* = 3.25, *p* < 0.05) and syntactic consistency (*β* = −5.43, *SE* = 0.29, *z* = −18.51, *p* < 0.001) were significant. The effect of IC was not statistically significant (*β* = 0.08, *SE* = 0.20, *z* = 0.40, *p* = 0.691). A marginal interaction was observed between plausibility and consistency (*β* = −0.72, *SE* = 0.39, *z* = −1.84, *p* = 0.066). More importantly, IC did not significantly modulate the effect of plausibility (*β* = −0.03, *SE* = 0.32, *z* = −0.10, *p* = 0.918), the effect of consistency (*β* = 0.17, *SE* = 0.26, *z* = 0.64, *p* = 0.522), or the interaction between plausibility and consistency (*β* = −0.12, *SE* = 0.41, *z* = −0.30, *p* = 0.761). These findings suggest that IC has no significant modulatory role under the positive valence condition.

**Figure 3:**
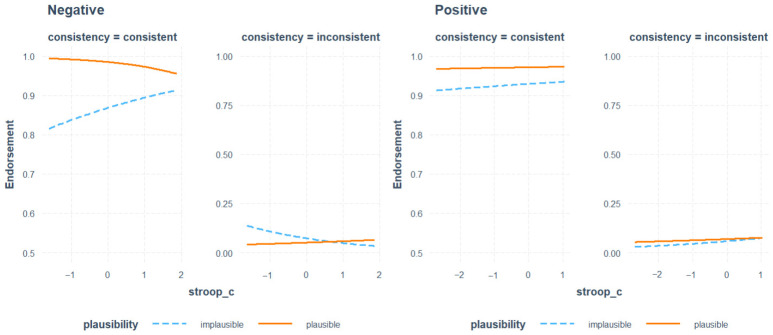
Endorsement by semantic plausibility and syntactic consistency and inhibitory control.

In the negative valence condition, IC showed a marginally significant effect (*β* = 0.23, *SE* = 0.13, *z* = 1.73, *p* = 0.085). It had significant interaction with plausibility (*β* = −0.79, *SE* = 0.36, *z* = −2.16, *p* < 0.05), and consistency (*β* = −0.63, *SE* = 0.22, *z* = −2.85, *p* < 0.05). Notably, we found a significant interaction between IC, plausibility, and consistency (*β* = 1.31, *SE* = 0.45, *z* = 2.91, *p* < 0.05). These results indicate that under negative affective states, IC influenced the relationship between plausibility and syntactic consistency. The relationship between the three variables in both groups is visually presented in Figure 3. As shown in the figure, the difference in the plausibility effect between syntactically consistent and inconsistent sentences was greater for participants with higher IC compared to low-IC participants. For higher-IC participants, the plausibility effect was large for consistent sentences (*β* = −3.16, *SE* = 0.77, *z* = −4.12, *p* < 0.001) but reduced for inconsistent ones (*β* = −0.95, *SE* = 0.45, *z* = 2.09, *p* = 0.037), with a variation of 4.11 log-odds units between conditions. For participants with lower IC, the plausibility effect was significant for consistent sentences (*β* = 1.45, *SE* = 0.36, *z* = −4.02, *p* < 0.001) but failed to reach significance in inconsistent ones (*β* = 0.19, *SE* = 0.34, *z* = −0.55, *p* = 0.583), yielding a smaller variation of 1.26 log-odds units. The interaction effect between consistency and plausibility was stronger among high-IC participants in the negative valence condition.

We also examined the role of working memory in relationships between mood and sentence comprehension. Before analysis, responses deviating more than 2.5 standard deviations from the means were discarded, which results in 2.18% of trials removed in negative valence and 3.30% removed in positive valence. Results revealed no significant influence of WM on participants’ responses (*β* = 0.20, *SE* = 0.16, *z* = 1.30, *p* = 0.193). The interaction between WM and syntactic consistency was marginally significant (*β* = −0.39, *SE* = 0.23, *z* = −1.73, *p* = 0.083). No other interactions involving WM reached significance, which shows that working memory did not modulate the relation between mood and sentence comprehension.

To assess whether WM effects were masked by reading behavior, we added log-transformed reading times for premises and conclusions as covariates to the model above. The RT-controlled model did not improve fit relative to the baseline WM model (ΔAIC = 3.9, *χ*^2^ = 0.093, *p* = 0.954). The main effect of WM and its interaction effects with other variables remained nonsignificant with estimates essentially unchanged. Thus, reading-time variance for these sentences does not account for the absence of WM effects.

### 3.4. Relationship Between RTs and Responses

Endorsement analysis alone can only reveal the final decisions regarding cue use in sentence processing; it cannot tell us how cue reliance varies with processing speed or the dynamic cognitive strategies underlying the decisions. Therefore, we further examined speed-response relationships to better understand the dynamic patterns of cue reliance. The purpose of the RT-response analysis is to understand the mechanisms and adaptability behind the endorsement patterns.

**Figure 4:**
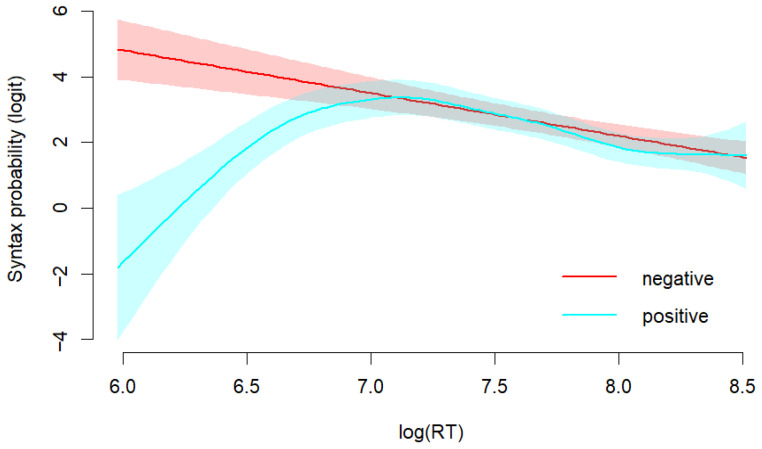
Proportion of syntax-based responses by RTs and group.

We analyzed the relationship between RTs and syntax-based responses, using a generalized additive mixed model. The model included fixed effects for experimental group, a smooth term for log-transformed RTs, and a factor-by-smooth interaction to test whether the RT-response relationship differed between groups. Random effects were included for subjects and items. Figure 4 presents the relationship between RTs and syntax-based responses across groups. The analysis revealed no significant influence of group on syntax probability (*β* = −0.21, *SE* = 0.18, *z* = −1.15, *p* = 0.250). The global RT effect was linear and significant (*edf* = 1.00, *χ*^2^ = 5.40, *p* < 0.05). Beyond this global trend, the relationship between RTs and responses differed across groups. For the negative-valence group, the effect of RTs on syntax was linear (*edf* = 1.00, *χ*^2^ = 10.09, *p* < 0.05). The positive-valence group showed a strong non-linear relationship (*edf* = 3.41, *χ*^2^ = 36.48, *p* < 0.001). In the positive valence condition, the proportion of syntax-based responses was low when RTs were short and increased to a high level when RTs increased. In the negative valence condition, the proportion of syntax-based responses remained relatively high regardless of RTs. The results show that positive and negative mood influence the use of syntactic cues in different ways. Participants in negative affective states consistently endorsed syntax-based interpretations even during rapid responses. However, those in positive affective states have barely used syntactic cues at very short times, but their syntactic reliance reached levels comparable to the negative group when RTs were longer. When participants had more time to respond, both groups relied on syntactic cues to understand sentences.

**Figure 5:**
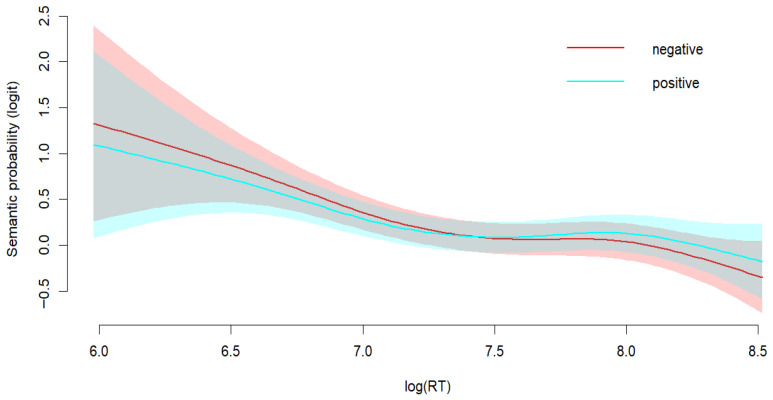
Proportion of semantics-based responses by RTs and group.

We also examined the relationship between RTs and semantics-based responses. Results showed that there was no significant overall group difference (*β* = −0.01, *SE* = 0.07, *z* = −0.09, *p* = 0.927). The overall smooth effect of RTs was significant (*edf* = 3.45, *χ*^2^ = 14.72, *p* = 0.007), suggesting a nonlinear relationship with responses. The group-specific smooth terms for RTs were not significant in the negative valence condition (*edf* = 0.24, *χ*^2^ = 0.04, *p* = 0.840), or the positive valence condition (*edf* = 1.00, *χ*^2^ = 0.44, *p* = 0.506), indicating that the effects of RTs was adequately captured by the overall smooth term in both groups. As shown in Figure 5, both groups relied more on semantic cues during fast responses and reduced their reliance when RTs increased.

## 4. Discussion

The present research investigated the influence of mood on the reliance on syntactic and semantic cues in sentence comprehension, as well as the modulation of such effect by inhibitory control and working memory. Participants completed a sentence judgment task, with one group exposed to a positive valence condition and another to a negative valence condition. The analysis of valence ratings confirmed a reliable difference across the two groups throughout the study, with participants in the positive valence condition scoring significantly higher than those in the negative valence condition.

Analysis of endorsement data has found both syntactic consistency and plausibility significantly influenced responses across groups, indicating that comprehenders made use of both cues regardless of their affective states. Interestingly, under the negative affective state, semantic plausibility interacted with syntactic consistency, while in the positive affective condition the two factors operated independently. This finding supports the research hypothesis based on the broaden-and-build theory (Fredrickson, 1998, 2001). Conversely, the hypothesis derived from the feelings-as-information theory (Schwarz, 2001; Beukeboom & Semin, 2006; Storbeck & Clore, 2008) is not supported. This indicates that mood shapes how semantic and syntactic cues are integrated, rather than which cue is preferred. One possible explanation is that individuals in a positive mood have greater attentional scope and cognitive flexibility (Fredrickson & Branigan, 2005). This flexibility enables them to accommodate multiple cues and shift more easily between syntactic and semantic cues during comprehension. These findings align with prior evidence that positive affect enhances cognitive flexibility (e.g., Nadler et al., 2010; Wang et al., 2017; Yang & Yang, 2014). They also support the Broaden Hypothesis within the framework of Fredrickson’s broaden-and-build theory (Fredrickson, 1998, 2001). Our analysis revealed a significant interaction between syntactic consistency and plausibility in negative mood. There are two possible reasons for such interaction. First, it might be related to the more rigid processing style and narrow focus of attention typically associated with negative affect. With narrow cognitive scope, comprehenders are likely to handle multiple cues interdependently. In this context, syntactic accuracy might be prioritized over semantic plausibility, due to task demands and the tendency of negative mood to promote analytical and algorithmic processing. This finding supports the Narrow Hypothesis which posits that negative affects narrow cognitive scope (Fredrickson & Branigan, 2005), making cognitive processes less flexible and more interdependent. This also supports previous studies showing that negative emotions lead to narrow scope of attention and reduced cognitive flexibility (e.g., Easterbrook, 1959; Finucane, 2011; Hsieh & Lin, 2019). Another reason is that negative mood fosters a more cautious and effortful processing style (Schwarz, 1990), which makes comprehenders adopt a secondary plausibility check when syntax is consistent. In other words, they are more vigilant about filtering out potentially misleading semantic cues. This makes it more likely for them to reject implausible statements, even when the syntax is consistent. When syntax is inconsistent, it is not necessary to use semantic cues as the statement is rejected outright. These findings advance the good-enough (GE) processing theory (Ferreira et al., 2002) by demonstrating how affective states modulate syntactic algorithm and semantic heuristics in sentence comprehension. In positive mood, GE processing occurred without cross-cue coupling, whereas in negative mood, it varied with syntactic conditions. This suggests that GE processing is not a static strategy but is instead dynamically shaped by affective contexts. While prior studies identified time pressure and task demands as conditions fostering GE processing (Ferreira et al., 2002; Swets et al., 2007), our results extend this repertoire, establishing affect as another critical factor that influences GE processing in emotionally charged settings.

To further explore the temporal dynamics of mood influence on sentence comprehension, we analyzed the relations between RTs and endorsement in different mood conditions. Interestingly, we found distinct patterns of RT-response relations between positive and negative moods. Participants in the positive mood relied less on syntactic cues at short RTs, and increased to levels comparable to the negative mood, whereas those in negative mood consistently relied on syntactic cues. This suggests a dynamic shift in processing strategy in positive mood, possibly driven by the broader cognitive scope (Fredrickson, 2001). Such strategy shift may be related to the task demand of this study. As the task required participants to judge sentences for logical consistency, it prompted them to make syntactic analysis. Therefore, participants may have shifted to syntax-based strategies to meet the task demand. In contrast, the negative-mood group had consistently high proportion of syntax-based responses across RTs, which reflects a stable, detail-oriented processing style. Negative mood enhanced focus on structural accuracy even under time constraints, which lends support to theoretical perspectives suggesting that negative mood promotes algorithmic and analytical cognitive processing style. We also found that both groups depended heavily on semantic plausibility during fast responses and reduced their reliance as RTs increased. The increased semantic reliance during fast responses reflects a compensation strategy. Participants rely on readily available semantic cues for quick responses as semantic heuristics offer a more efficient way to obtain meaning. As RTs increased, they shifted to a more syntax-driven approach to meet the task demand. The RT patterns cannot be explained by either purely parallel or purely sequential models. Instead, the data are compatible with accounts in which multiple cues are activated in parallel but reweighted over time. We therefore interpret the findings as evidence for dynamic cue weighting shaped by mood and task demands. At the same time, because RT patterns can sometimes resemble sequential effects, more time-sensitive measures such as EEG or eye tracking are needed to more clearly distinguish the dynamic cue weighting account from the sequential processing account.

This study also examined the role of cognitive factors including WM and IC in the mood-by-language interactions. The results indicated that WM did not significantly affect sentence comprehension, nor did it modulate the mood effect. A marginal interaction between WM and syntactic consistency suggested that participants with larger WM might rely more on syntactic analysis during sentence comprehension. However, this tendency failed to reach statistical significance, possibly because our experimental task was not cognitively demanding enough to challenge participants’ WM capacity. As the sentence judgement task is relatively simple, participants may not have needed to use a lot of their WM resources. As Just and Carpenter (1992)’s total capacity hypothesis posits, WM capacity would only influence performance when the task requires more resources than are available. Another possible reason for the nonsignificant WM effect is that this study adopted the digit span task which measures general WM capacity, rather than language-specific WM span. Unlike measures like reading span (Daneman & Carpenter, 1980), this task may not be sensitive to the specific WM demands of syntactic or semantic processing, as it primarily measures information storage rather than the complex processing required for sentence comprehension. Similar tendency was also reported in Obler et al. (1991)’s study which found that WM, as measured by the digit span task, had little effect on sentence comprehension. Further research should consider using tasks with a higher WM load and language-specific WM measures such as reading span to better capture the role of WM in affect-driven sentence comprehension. Another possible reason for the lack of WM effect is related to mood-related disengagement. Previous studies have found that positive and negative mood can sometimes lead to disengagement (e.g., Fredrickson, 2001), though for different reasons. For instance, as positive mood increases reliance on heuristics and reduces effortful processing (Fredrickson, 2001), people in positive mood are likely to withdraw efforts and thus, not fully exploit their WM resources. Such disengagement might dilute the role of WM in cognitive activities. Furthermore, affective states may have influenced the use of linguistic cues through other executive function components, such as inhibitory control, potentially overshadowing the WM effect (Miyake et al., 2000).

Compared with WM, inhibitory control played a more significant role in the mood-by-language interaction. It was found that the interaction between syntactic consistency and semantic plausibility was stronger among high-IC participants in the negative mood. This might be because that comprehenders with higher IC prioritize syntactic analysis and suppress heuristic reliance on plausibility. As plausibility-based heuristics can sometimes lead to incorrect interpretation, high-IC participants inhibited premature judgments, ensuring semantic plausibility was evaluated only if syntactic correctness was confirmed. The finding is supported by behavioral and neurological evidence showing inhibitory functions centered in the prefrontal cortex are essential for shifting from heuristic and habitual behaviors to controlled behaviors (Anderson & Weaver, 2009; Hwang et al., 2014; Wiecki & Frank, 2013). In a positive mood, IC was not found to influence the use of linguistic cues. It may be because the flexible, inclusive processing style associated with positive affect (Fredrickson & Branigan, 2005) makes suppression less relevant. Semantic plausibility is allowed to contribute to comprehension regardless of its alignment with syntactic structure, and strong IC is not required to suppress it. Prior research has shown that executive functions (EF) facilitate sentence comprehension by resolving ambiguities, integrating syntactic and semantic cues, and managing cognitive demands (Miyake et al., 2000; Cutting et al., 2009; Sesma et al., 2009). This study extends previous findings to show how IC, as an EF component, dynamically adapts sentence comprehension to affective contexts. This adds a novel dimension to the field and highlights the need for psycholinguistic models that integrate EF modulation to explain mood-driven cue reliance.

Findings from this study have major theoretical and practical implications. Traditional psycholinguistic accounts often treat linguistic processing as an affect-free cognitive operation. However, people continuously experience different kinds of affect in everyday communication. By showing that mood and inhibitory control shape the interpretation of language, the present study highlights the need for integrative models of sentence processing that incorporate both affective and executive mechanisms. This study has practical significance across diverse real-world contexts, where mood and cognitive capacities may systematically influence comprehension outcome. In education, language learners’ affective states could interact with executive functions to modulate how they comprehend complex sentences. Instructional strategies need to be tailored to learners’ affective and cognitive profiles to optimize learning outcome. Our findings also offer new perspectives for interventions in clinical populations. Individuals with depression or anxiety often experience negative mood states. Such patients may have difficulty interpreting language with conflicting syntactic and semantic cues, due to their narrow cognitive scope. Interventions that foster positive mood such as relaxation training or music therapy could help reduce their comprehension difficulties. Meanwhile, clinicians and therapists should consider adapting their communication by using unambiguous language to ease the cognitive burdens for patients.

This study is not without limitations. As shown in the analysis, this study did not find WM effects on sentence comprehension. This might be because the experimental task is not cognitively demanding enough to fully engage WM resources. Future studies could employ tasks with greater memory load to assess the role of WM in language comprehension. Second, this study mainly relies on an offline sentence judgment task, which assesses cue use analogically through post-comprehension decisions rather than directly capturing real-time sentence processing. Online measures, such as eye-tracking or ERPs, provide finer-grained insights into the dynamics of syntactic and semantic processing. Future research could employ these methods to validate whether the endorsement patterns observed here in this study reflect real-time comprehension processes. Third, the film clips used for positive mood induction were not effective in enhancing the baseline valence levels in the positive-mood group, which indicates that the induction was not successful. Future research should consider using stronger or alternative induction methods, such as personalized stimuli tailored to individual preferences, multi-modal approaches combining music and image, or repeated induction sessions, to ensure successful manipulation of positive mood. Such improvements could increase the effectiveness of mood manipulations and provide clearer insights into the effects of induced mood on sentence comprehension. Another limitation is that we only looked at positive and negative moods. It is not clear how a neutral mood or other affective dimensions such as arousal might contribute to sentence comprehension. More research is needed to explore whether our findings generalize across a broader affective context. Beyond induced moods, the intrinsic valence of language at the word or sentence level may also shape the use of linguistic cues, possibly via mood-congruent processing. Future studies should systematically vary experimental materials to assess whether stimulus valence contributes to participants’ use of syntactic or semantic cues in language comprehension.

## Figures and Tables

**Table 1:** Demographic information.

	Positive	Negative
Age	23.1/1.70 (21−28)	23.1/2.36 (19−31)
Education (years)	17.2/1.64	16.9/2.51
Gender (Male %)	38.9%	42.1%

**Table 2:** Sample experimental sentences.

Condition	Sentence	Probe
CP	a. nage xunlian qiuyuan de jiaolian jianchale qicai	The coach trained the athletes.
	b. the trained athletes DE coach checked equipment	
	c. The coach who trained the athletes checked the equipment.	
CImp	a. nage xunlian jiaolian de qiuyuan jianchale qicai	The athletes trained the coach.
	b. the trained coach DE athletes checked equipment	
	c. The athletes who trained the coach checked the equipment.	
IncP	a. nage xunlian jiaolian de qiuyuan jianchale qicai	The coach trained the athletes.
	b. the trained coach DE athletes checked equipment	
	c. The athletes who trained the coach checked the equipment.	
IncImp	a. nage xunlian qiuyuan de jiaolian jianchale qicai	The athletes trained the coach.
	b. the trained athletes DE coach checked equipment	
	c. The coach who trained the athletes checked the equipment.	

Notes: a. original sentence in pinyin; b. English gloss; c. English translation.

**Table 3:** Descriptive statistics for accuracy and reaction times by group and condition.

Group	Consistency	Plausibility	Accuracy (M/SD)	RT (M/SD)
Negative	Consistent	Plausible	0.981 (0.136)	1.590 (0.758)
Negative	Consistent	Implausible	0.908 (0.289)	1.878 (0.864)
Negative	Inconsistent	Plausible	0.947 (0.224)	1.906 (0.875)
Negative	Inconsistent	Implausible	0.945 (0.227)	1.935 (0.909)
Positive	Consistent	Plausible	0.964 (0.186)	1.576 (0.764)
Positive	Consistent	Implausible	0.915 (0.280)	1.795 (0.832)
Positive	Inconsistent	Plausible	0.911 (0.285)	1.872 (0.864)
Positive	Inconsistent	Implausible	0.937 (0.244)	1.782 (0.801)

## Data Availability

The data presented in this study are available on request from the authors.
